# Prognostic Significance of Activated Leukocyte Cell Adhesion Molecule (ALCAM) in Association with Promoter Methylation of the *ALCAM* Gene in Breast Cancer

**DOI:** 10.3390/molecules23010131

**Published:** 2018-01-09

**Authors:** Young Ju Jeong, Hoon Kyu Oh, Sung Hwan Park, Jin Gu Bong

**Affiliations:** 1 Department of Surgery, School of Medicine, Catholic University of Daegu, Daegu 42471, Korea; yjjeong@cu.ac.kr (Y.J.J.); shwpark@cu.ac.kr (S.H.P.); 2 Department of Pathology, School of Medicine, Catholic University of Daegu, Daegu 42471, Korea; ap510@cu.ac.kr

**Keywords:** activated leukocyte cell adhesion molecule, methylation, inflammation, epigenetics, breast cancer

## Abstract

Activated leukocyte cell adhesion molecule (ALCAM) has been implicated in tumorigenesis. In this study, we studied DNA methylation status of the *ALCAM* gene using pyrosequencing in breast cancer tissues. We analyzed the association between the methylation status of the *ALCAM* gene and its expression. Also, the effects of inflammation on the *ALCAM* gene methylation and its expression were investigated. The *ALCAM* gene methylation was associated with the *ALCAM* transcripts in tumor tissues. The methylation status of the *ALCAM* gene was not significantly different between tumor and normal tissues. The level of *ALCAM* transcripts was associated with the expression of TNFα, NF-κB p50, IL-4, and intratumoral inflammation. The IHC expression of ALCAM was associated with histologic grade, HER2 overexpression and molecular subtype. The expression of TNFα, NF-κB p50, and IL-4 showed significant association with the clinicopathologic characteristics. In conclusion, the *ALCAM* gene methylation was related to the level of *ALCAM* transcripts. Also, the level of *ALCAM* transcripts was associated with the inflammatory markers in breast cancer. Our results suggest that the methylation of the *ALCAM* gene contributes to the decreased expression of ALCAM. Also, ALCAM is linked to the inflammatory response in breast cancer.

## 1. Introduction

Cellular adhesion molecules (CAMs) are cell-surface proteins that mediate cell-to-cell interaction and interaction between cell and extracellular matrix [1]. CAMs are important in cell growth, cell migration, and cell differentiation [2], and affect the cellular communications, the signal transduction, and inflammatory responses [3]. In recent years, CAMs have been implicated in tumorigenesis and tumor metastasis [1,2,4]. CAMs can be divided into for major groups, including cadherins, integrins, selectins, and the immunoglobulin superfamily [1].

Activated leukocyte cell adhesion molecule (ALCAM), also known as CD166, is a transmembrane glycoprotein that is a member of the immunoglobulin superfamily with five immunoglobulin-like domains [5]. It is encoded by the *ALCAM* gene on chromosome 3 in region 3q13.1–q13.2 [5]. ALCAM is expressed on epithelial, endothelial, neuronal, and hematopoietic cells [5,6,7]. ALCAM is one of CAMs and is involved in cell adhesion as well as in embryogenesis, angiogenesis, hematopoiesis, osteogenesis, neural cell migration, and immune response [6,7]. In recent years, alterations in ALCAM expression have been reported in various malignancies, such as melanoma, cancer in lung, esophagus, colon, pancreas, prostate, bladder, ovary, and breast [8,9,10,11,12,13,14,15,16]. 

In breast cancer, most of studies described that reduced ALCAM expression indicates a more aggressive phenotype and poor prognosis [16,17,18,19], whereas several studies showed that increased ALCAM expression is associated with poor prognosis [20,21,22]. Several mechanisms can be suggested to explain these conflicting results. For example, ALCAM expression at the cell surface can be modulated by specific ligand-induced interaction [23]. Also, ALCAM expression in tumor cells can be downregulated by DNA methylation [24].

Epigenetic modification of a gene, especially promotor methylation of specific gene loci is a well-established mechanism leading to gene silencing. However, only a few studies have analyzed the methylation status of the *ALCAM* gene [24], and the mechanisms that regulate the *ALCAM* gene expression are not well defined. The purpose of this study was to investigate epigenetic alterations of the *ALCAM* gene using pyrosequencing analysis and to analyze the association between the methylation of the *ALCAM* gene and its expression in breast cancer. We hypothesized that epigenetic alterations of the *ALCAM* gene affect ALCAM expression in relation to inflammation and contribute to the progression of breast cancer. We also analyzed prognostic significances of the *ALCAM* gene methylation and its expression.

## 2. Results

### 2.1. Clinicopathologic Characteristics

The average age of the patients was 55.77 ± 13.47 years. The mean tumor size was 1.97 ± 1.04 cm (range, 0.1–4.5 cm). Among the 47 patients studied, 38.3% of the patients showed metastasis to regional lymph nodes. Eighteen patients (38.3%) had stage I disease, 24 patients (51.0%) stage II, 3 patients (6.4%) stage III, and two patients (4.3%) stage IV. According to the clinical classification of intrinsic subtype in breast cancer [25], 9 patients (22.0%) were luminal A subtype, 23 patients (56.1%) were luminal B subtype, 6 patients (14.6%) were human epidermal growth factor receptor 2 (HER2) subtype and three patients (7.3%) were basal-like subtype. 

### 2.2. Association of Methylation Status of the ALCAM Gene and ALCAM Expression

The overall mean methylation level of the *ALCAM* gene was 3.41 ± 4.56%. Among 47 tumor tissues, 95.6% of the cases showed aberrant methylation of the *ALCAM* gene. The mean methylation level of the *ALCAM* gene was higher in tumor tissues than that of normal tissues (3.55 ± 4.95% and 2.74 ± 2.19%), but there was no statistically significant difference (*p* = 0.612). We analyzed the expression of ALCAM by reverse transcriptase polymerase chain reaction (RT-PCR) of RNA transcripts in frozen tissues, as well as by IHC staining on TMA tissue sections. In the analysis of *ALCAM* transcripts, the level of the *ALCAM* gene methylation was significantly higher in negative *ALCAM* transcripts group in tumor tissues (*p* = 0.027) (Table 1). In the analysis of IHC expression, the level of the *ALCAM* gene methylation was relatively lower in strong positive ALCAM expression group when comparing with other groups, but no statistical significances were shown (*p* = 0.285, data not shown). According to the results of RT-PCR of *ALCAM* transcripts, the level of ALCAM transcripts was not significantly different between tumor and normal tissues (*p* = 0.716, data not shown).

### 2.3. Association of Methylation Levels of the ALCAM Gene and ALCAM Expression with Inflammatory Markers in Tumor Tissues

The level of *ALCAM* transcripts was positively associated with the expression of tumor necrosis factor α (TNFα), nuclear factor-kappa B (NF-κB) p50 subunit, and interleukin (IL)-4 in tumor tissues (*p* < 0.001, *p* < 0.001 and *p* = 0.012, respectively). On the other hand, the level of *ALCAM* transcripts showed negative association with intratumoral inflammation (*p* = 0.041) (Table 2). The methylation status of the *ALCAM* gene was not significantly associated with inflammatory markers. The immunohistochemical (IHC) expression of ALCAM was not significantly associated with inflammatory markers although the relation patterns were similar to those of *ALCAM* transcript (data not shown).

**Figure 1:**
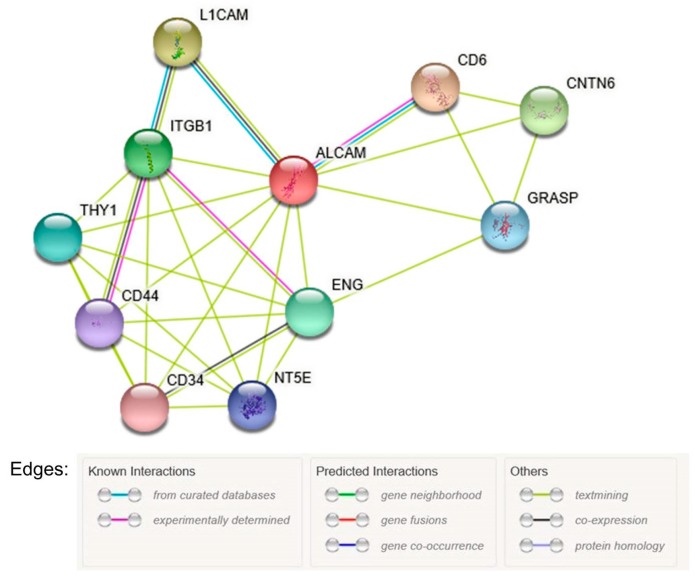
Protein-protein interaction network of ALCAM constructed with STRING version 10.5, ALCAM, activated leukocyte cell adhesion molecule.

To analyze functional relationship between ALCAM expression and inflammatory markers, we used Search Tool for the Retrieval of Interacting Genes (STRING) and the Biological General Repository for Interaction Datasets (BioGRID). In the protein-protein interaction (PPI) network, there were 11 nodes and 30 interactions (Figure 1). The predicted functional partners were CD6, L1CAM, CNTN6, ITGB1, ENG, Thy1, GRASP, NT5E, CD44, and CD34. Among the proteins in the PPI network, CD6 is a cell adhesion molecule that regulates T-cell responses via its interaction with ALCAM [26].

### 2.4. Association of Methylation Levels of the ALCAM Gene and Its Expression with the Clinicopathologic Characteristics

The IHC expression of ALCAM showed negative association with histologic grade and HER2 overexpression (*p* = 0.015 and *p* = 0.021, respectively) (Table 3). The IHC expression of ALCAM was also associated with molecular subtype of breast cancer (*p* = 0.001) and showed most strong expression in Luminal A subtype. However, there was no significant association between clinicopathologic characteristics and the mean methylation level of the *ALCAM* gene, as well as the level of *ALCAM* transcripts in this study.

### 2.5. Association between Inflammatory Markers and the Clinicopathologic Characteristics in Breast Cancer

We analyzed the relationship between inflammatory markers and breast cancer. The expression of TNFα showed significant association with histologic grade, molecular subtype, HER2 overexpression, and Bcl 2 expression (*p* = 0.039, *p* = 0.011, *p* = 0.037, and *p* = 0.041, respectively) (Table 4). The expression of NF-κB p50 subunit showed significant association with estrogen receptor (ER) and molecular subtype (*p* = 0.046 and *p* = 0.022, respectively) (Table 5). The expression of IL-4 was significantly associated with tumor size, stage, lymphovascular invasion, and lymph node metastasis (*p* = 0.029, *p* = 0.040, *p* = 0.003 and *p* = 0.002, respectively) (Table 6).

### 2.6. Discussion

Aberrant DNA methylation is an important mechanism of tumor development and progression [27]. In breast cancer, a number of tumor suppressor genes are known to be inactivated by promoter hypermethylation [28]. However, there are only a few studies to analyze the methylation status of the *ALCAM* gene, although ALCAM has been implicated in prognosis of breast cancer [16,17,18,19,20,21,22]. King et al. [24] studied the mechanisms of transcription regulation of *ALCAM* using cell lines and showed that the *ALCAM* promoter was methylated in MDA-MB-435 cell lines that lack ALCAM expression. Consistent with the results of previous study, our study also showed that the level of the *ALCAM* gene methylation is associated with the levels of *ALCAM* transcripts in tumor tissues. In this regard, promoter hypermethylation of the *ALCAM* gene is thought to downregulate transcription in tumor tissues. To our knowledge, this study represents the first investigation to analyze the promoter methylation status of the *ALCAM* gene in human breast cancer tissues using pyrosequencing. In addition, this is one of a few studies that revealed the association between ALCAM expression and the *ALCAM* gene methylation in breast cancer.

Interestingly, the association between the *ALCAM* gene methylation and the levels of *ALCAM* transcripts was only significant in tumor tissues, but not normal tissues in this study. The mechanisms related to our findings are not elucidated, but a possible mechanism is a reversible change in DNA methylation. According to the previous study, targets of DNA demethylation and proteins for DNA demethylase activity are different between normal and cancerous cell lines [29]. Recently, Jeong et al. reported the reversibility of DNA methylation by integrative analysis of DNA methylation and mRNA expression [30]. Baysan et al. described that DNA methylation and mRNA transcription undergo significant and reproducible transformation from in vitro to in vivo growth conditions and then back again [31]. They suggested that microenvironment causes reversible changes in DNA methylation and mRNA expression. It is widely recognized that tumor microenvironment is different from the microenvironment in normal tissues and is closely connected to tumorigenesis [32]. Therefore, unlike tumor tissues, reversible changes of DNA methylation can affect mRNA expression differently in normal tissues. This hypothesis needs to be validated with further study.

Meanwhile, ALCAM is involved in immune response, although the precise immunological mechanisms in breast cancer have yet to be elucidated. Zimmerman et al. [33] demonstrated that heterotypic interactions between ALCAM and T-cell antigen CD6 is essential for T-cell proliferation. A recent report by Kudo-Saito et al. [34] showed that immunoregulatory ALCAM-positive cells prevent generation of potent cytotoxic T lymphocytes via ALCAM, leading to tumor progression after cryoablation in cancer. King et al. [24] also reported that over-expression of p65 NF-κB increased *ALCAM* promoter activity, suggesting that ALCAM is a target of the NF-κB pathway. Nevertheless, most studies described experimental results, and the clinical relevance remains to be established. In this study, we analyzed the association between ACLAM and inflammation using clinical data. Our study showed that positive *ALCAM* transcripts were related to suppression of intratumoral inflammation. Also, the level of inflammatory markers including TNFα, NF-κB p50 subunit and IL-4 in tumor tissues showed positive correlation with the level of *ALCAM* transcripts. Therefore, our results also suggest that ALCAM is involved in the immunologic response to tumor cells.

For recent years, it has been indicated that low expression of ALCAM is a poor prognostic marker in breast cancer [16,17,18,19]. Our study also showed that reduced ALCAM expression was associated with high histologic grade, HER2 overexpression, and molecular subtype. Interestingly, in a recent study investigating DNA methylation profiling in breast cancer, the authors revealed that the high expression of epigenetically regulated immune genes were associated with a better clinical outcome [35]. From our results, *ALCAM* is thought to be regulated epigenetically and has a possibility of immune related gene. In this regard, it can be assumed that increased ALCAM expression is associated with good prognosis, although the methylation status of the *ALCAM* gene was not included in previous study [36].

For decades, the role of inflammation in breast cancer has become increasingly obvious. Immune cells such as macrophages and tumor infiltrating lymphocytes (TIL) are known to be related to breast cancer prognosis [35,37]. Especially, the presence of TILs is a favorable prognostic factor in breast cancer [38]. TIL in breast cancer can be used to predict to therapy [39]. Also, increased TIL has been associated with increased PD-L1 infiltrate and lymphocyte-predominant breast cancers can be treated with immune therapy, such as immune check point inhibitor, anti-PD-L1 monoclonal antibody [40]. Interestingly, our study showed conflicting results that positive ALCAM expression was negatively associated with intratumoral inflammation, but was related to better clinicopathologic characteristics. However, there was no direct association between intratumoral inflammation and clinicopathologic characteristics. Proinflammatory cytokines and chemokines such as IL-1, IL-6, IL-8, and TNFα, and NF-κB family transcription factors are also recognized as important factors in inflammation and breast cancer [41]. In this study, the expressions of TNFα, NF-κB p50 subunit, and IL-4 were related to specific clinicopathologic characteristics in breast cancer.

In conclusion, we showed that the frequency of the *ALCAM* gene methylation was negatively related to the level of *ALCAM* transcripts. The result suggests that the methylation of the *ALCAM* gene contributes to the decreased expression of ALCAM. This study indicates that the methylation of *ALCAM* genes regulates the level of expression. Also, the level of *ALCAM* transcripts was associated with the expression of TNFα, NF-κB p50, IL-4, and intratumoral inflammation in breast cancer. ALCAM and inflammation has been related with breast carcinogenesis. So, there is possible link between ALCAM expression and the inflammatory response in breast cancer. Furthermore, the level of ALCAM expression was positively associated with better clinicopathologic features. Taken together, our study showed that ALCAM has potential utility as a novel prognostic and predictive biomarker. Further studies are needed to clarify the relevance of the epigenetic mechanism in the regulation of the ALCAM expression and prognostic value of ALCAM in breast cancer.

## 3. Materials and Methods

### 3.1. Patients and Materials

Among the patients with breast cancer who underwent surgery between May 2008 and July 2012 at Daegu Catholic University Hospital (Daegu, South Korea), a total of 47 patients who presented invasive ductal carcinoma were included in this study. The small pieces of samples from breast cancer tissues were collected in sterile collection tubes immediately after the surgery and then stored in −80 °C until further analysis. All of the specimens except frozen tissues were fixed in formalin and embedded in paraffin, then stained with hematoxylin and eosin (H & E) for histologic examination. The specimens were reviewed by an experienced pathologist. Normal mammary tissues were obtained from the formalin-fixed, paraffin-embedded non-neoplastic breast tissue specimens in 10 patients. The clinicopathologic characteristics were evaluated from the medical records. Ethics approval for the study was obtained from the Institutional Review Board at Daegu Catholic University Hospital.

### 3.2. DNA Extraction and Methylation Analysis

**Figure 2:**
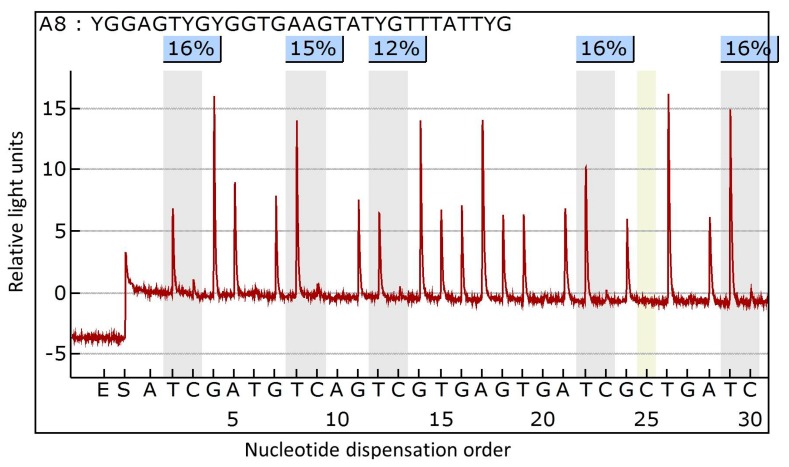
Representative pyrogram of analyzing methylation status of the *ALCAM* gene using pyrosequencing. The sequence in the upper part of the pyrogram represents the sequence to be analyzed. The gray regions indicate the analyzed C/T sites, with percentage values for the respective cytosine above them. The yellow region indicates the parts where a cytosine was added to verify the complete conversion from unmethylated cytosine to thymine.

The fresh frozen primary breast cancer tissues were prepared for DNA extraction. Genomic DNA was isolated using the QIAamp DNA Mini Kit (Qiagen, Hilden, Germany) following the manufacturer’s instructions. The purified DNA was quantified using a ND-1000 spectrophotometer (NanoDrop Technologies Inc., Wilmington, DE, USA). The quality of the DNA was verified by gel electrophoresis. Sodium bisulfate modification of 200 ng genomic DNA was performed using the EZ DNA Methylation-Lightning kit (Zymo Research, Orange, CA, USA), according to the manufacturer’s protocol. For polymerase chain reaction (PCR) of *ALCAM* gene, forward primer was 5′-GGTGGGTAGTTGGAAGTTAGAG-3′ and reverse primer was 5′-AAATCCCAACCCTAAACCCAACTC-3′. PCR was performed using bisulfate-treated DNA under the following conditions: 95 °C for 10 min; 45 cycles of 95 °C for 30 s, 58 °C for 30 s and 72 °C for 30 s; and, final extension of 5 min at 72 °C. Methylation analysis of the *ALCAM* gene was performed by pyrosequencing using PyroGold reagent kit and PyroMark ID (Qiagen, Hilden, Germany). Primer for DNA sequencing was 5′-GATTTGGTTTTGGGGG-3′. We examined five CpG sites in the promoter region of *ALCAM* gene. The methylation index (MtI) of *ALCAM* gene in each sample was calculated as the average value of ^m^*C*/(^m^*C* + *C*) for all of the examined CpGs in target regions. The representative example of pyrogram is shown in Figure 2. All the experiments included a negative control without template.

### 3.3. RNA Extraction and Analysis of ALCAM Transcripts

**Figure 3:**
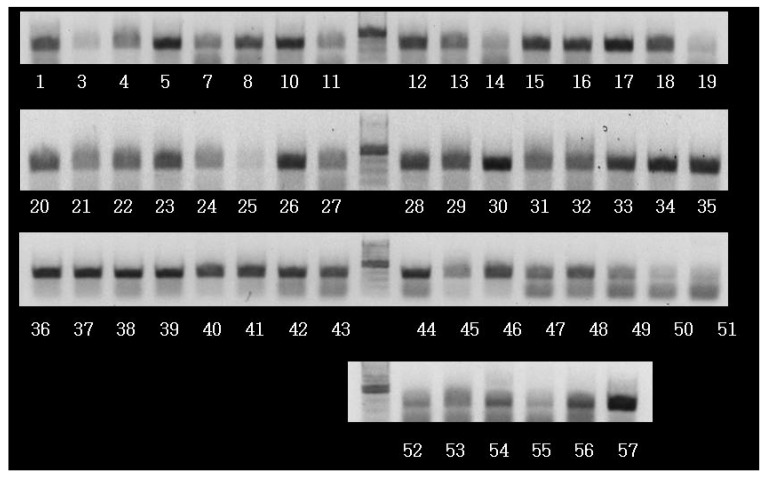
Result of electrophoresis of ALCAM transcripts after reverse transcriptase polymerase chain reaction (RT-PCR).

The levels of ALCAM, TNF-α, IL-2, IL-4, IL-6, interferon-γ, NF-κB p50, β-catenin, E-cadherin, N-cadherin, and M-CSF were assessed by the levels of RNA transcripts in frozen tissues using RT-PCR. Total RNA was extracted from frozen breast cancer tissues using Trizol reagent (#A33250; Invitrogen (Carlsbad, CA, USA); Thermo Fisher Scientific, Inc., Waltham, MA, USA), according to the manufacturer’s protocol. Reverse transcription of total RNA was performed using a commercial kit (Superscript II RNase H-reverse transcriptase; #18064071; Invitrogen; Thermo Fisher Scientific, Inc.). PCR products were analyzed by agarose gel electrophoresis and visualized by ethidium bromide staining (Figure 3). For PCR of ALCAM transcripts, forward primer was 5′-CAAGACAACCAAGGCTGACA-3′ and reverse primer was 5′-CGCAGACATAGTTTCCAGCA-3′. The level of ALCAM transcripts was analyzed using Quantity One^®^ 1-D Analysis Software (Bio-Rad Laboraties, Inc., Hercules, CA, USA). The expression of inflammatory markers were assessed semiquantitatively as negative (no band), weak positive (weak band), and strong positive (strong band). The levels of expression were classified as positive for any expression and negative for no expression.

### 3.4. Tissue Microarray and Immunohistochemistry

Representative paraffin embedded primary breast cancer tissues were selected and prepared for tissue microarray (TMA) construction. TMA was constructed following the methods described in our previous study [42]. IHC staining for ALCAM, E-cadherin, and other prognostic markers of breast cancer were performed on 5 µm-thick TMA sections using the Bond Polymer Intense Detection System (Leica Microsystems, Victoria, Australia). We used commercially available primary antibodies for IHC staining as below: ALCAM (1:450, clone MOG/07; Novocastra, Newcastle, UK), E-cadherin (1:50, clone 36B5; Novocastra), ER (ER; #NCL-L-ER-6F11; 1:100; clone 6F11; Leica Biosystems, Newcastle, UK), progesterone receptor (PR; #NCL-L-PGR-312; 1:100; clone 16; Novocastra; Leica Biosystems), HER2 (HER2; #A048529-1; 1:250; clone #A0485; Dako; Agilent Technologies, Inc., Santa Clara, CA, USA), Ki-67 (Ki-67; #275R-16; 1:200; clone MM1-L; Cell Marque; Sigma-Aldrich Co., LLC, Darmstadt, Germany), Bcl-2 (Bcl-2; #IR614; 1:4; clone 124; Dako; Agilent Technologies, Inc.), p53 (p53; #18-0129; 1:200; clone BP53.12; Invitrogen; Thermo Fisher Scientific, Inc.) and epidermal growth factor receptor (EGFR; #M7239; 1:100, clone EGFR.25; Dako; Agilent Technologies, Inc.).

The levels of ALCAM expression were assessed semiquantitatively as 0 (no staining), 1+ (weak, <10% of cells), 2+ (moderate, 10–49%), and 3+ (strong, ≥50%). Intratumoral (within the tumor boundary) and peritumoral (at the edge of tumor boundary) lymphocyte infiltration was assessed semiquantitatively as 0 (no or scant lymphocytes), 1 (a few scattered lymphocytic infiltration), 2 (scattered lymphocytic aggregation), and 3 (diffuse and dense aggregation of lymphocytes). The levels of intratumoral and peritumoral inflammation were classified as positive for 1, 2, and 3, and negative for 0.

### 3.5. Bioinformatic Analysis

To analyze the functional relationship between ALCAM and inflammatory molecules, we used STRING Database (http://www.string-db.org/) and BioGRID Database (http://thebiogrid.org/). The protein-protein interaction (PPI) network was constructed using STRING version 10.5 [43]. Interactions with a combined score >0.4 were selected as significant.

### 3.6. Statistical Analysis

Statistical analyses were performed using SPSS software version 19.0 (SPSS, Inc., Chicago, IL, USA). Association between the methylation status of the *ALCAM* gene and its expression was assessed using two sample *t*-test or non-parametric Mann-Whitney U test. A comparison of the mean methylation frequencies between tumor and normal tissues was performed using two sample *t*-test or non-parametric Mann-Whitney U test. Associations between the *ALCAM* gene methylation status and the clinicopathologic characteristics were assessed using two sample *t*-test or the non-parametric Mann-Whitney U test for categorical variables, and correlation between 2 continuous variables was assessed using correlation analysis. The relationship between ALCAM expression and the clinicopathologic characteristics of the patients was analyzed using the Chi-square test or the Fisher’s exact test for categorical data and two sample *t*-test or the non-parametric Mann-Whitney U test for continuous data. All of the tests were two-sided and a *p*-value of <0.05 was considered to indicate a statistically significant difference.

## Figures and Tables

**Table 1:** Association of methylation levels of the activated leukocyte cell adhesion molecule (*ALCAM*) gene and its expression in tumor tissues and normal breast tissues.

		*ALCAM* Methylation Levels			
		Tumor		Normal	
		Mean (%)	*p*-Value	Mean (%)	*p*-Value
ALCAM transcript	Negative	5.17 ± 6.83	0.027	2.07 ± 0.52	0.951
	Positive	2.66 ± 3.34		2.04 ± 0.76	

ALCAM, activated leukocyte cell adhesion molecule.

**Table 2:** Association of methylation levels of the *ALCAM* gene and its expression with other inflammatory markers in tumor tissues.

Inflammatory Markers		*ALCAM* Methylation		ALCAM Transcripts	
		Mean Levels (%)	*p*-Value	Positive Expression, n (%)	*p*-Value
TNF-α	Negative	3.11 ± 3.41	0.190	6 (37.5)	<0.001
	Positive	3.50 ± 5.30		24 (77.4)	
NF-κB p50	Negative	9.27 ± 10.51	0.318	3 (16.7)	<0.001
	Positive	3.12 ± 4.31		10 (76.9)	
IL-4	Negative	4.20 ± 5.74	0.234	5 (41.7)	0.012
	Positive	2.51 ± 3.04		19 (90.5)	
Intratumoral inflammation	Negative	5.23 ± 8.87	0.429	7 (87.5)	0.041
	Positive	3.47 ± 4.22		17 (60.7)	

ALCAM, activated leukocyte cell adhesion molecule.

**Table 3:** Association between the clinicopathologic characteristics and the ALCAM IHC results in tumor tissues.

Clinicopathologic Characteristics	ALCAM IHC Results				
	Negative (%)	Weak (%)	Moderate (%)	Strong (%)	*p*-Value
Histologic grade					0.015
1	0.0	33.3	16.7	50.0	
2	9.1	27.3	36.4	27.3	
3	29.4	35.3	29.4	5.9	
HER2 overexpression					0.021
Negative	6.7	20.0	40.0	33.3	
Positive	20.0	46.7	26.7	6.7	
Molecular Subtype					0.001
Luminal A	0.0	0.0	16.7	83.3	
Luminal B	22.2	38.9	38.9	0.0	
HER2	0.0	50.0	25.0	25.0	
Basal-like	0.0	50.0	50.0	0.0	

ALCAM, activated leukocyte cell adhesion molecule; HER2, human epidermal growth factor receptor 2.

**Table 4:** Association between tumor necrosis factor α (TNFα) and clinicopathologic characteristics in breast cancer.

Clinicopathologic Characteristics		TNFα Expression			
		Negative (%)	Weak Positive (%)	Strong Positive (%)	*p*-Value
Histologic grade	1	25.0	12.5	62.5	0.039
	2	33.3	50.0	16.7	
	3	37.0	18.5	44.4	
Molecular subtype	Luminal A	33.3	33.3	33.3	0.011
	Luminal B	26.1	30.47	43.5	
	HER2	83.3	0.0	16.7	
	Basal-like	33.3	0.0	66.7	
HER2 overexpression	Negative	30.4	17.4	52.2	0.037
	Positive	44.4	33.3	22.2	
Bcl 2 expression	Negative	50.0	0.0	50.	0.041
	Positive	30.8	30.8	38.5	

HER2, human epidermal growth factor receptor 2.

**Table 5:** Association between nuclear factor-kappaB (NF-κB) and clinicopathologic characteristics in breast cancer.

Clinicopathologic Characteristics		NF-κB Expression			
		Negative (%)	Weak Positive (%)	Strong Positive (%)	*p*-Value
ER	Negative	18.2	54.5	27.3	0.046
	Positive	12.0	28.0	60.0	
Molecular subtype	Luminal A	25.0	37.5	37.5	0.022
	Luminal B	11.8	17.6	70.6	
	HER2	25.0	75.0	0.0	
	Basal-like	0.0	50.0	50.0	

ER, estrogen receptor; HER2, human epidermal growth factor receptor 2.

**Table 6:** Association between interleukin (IL-4) and clinicopathologic characteristics in breast cancer.

Clinicopathologic Characteristics		IL-4 Expression			
		Negative (%)	Weak Positive (%)	Strong Positive (%)	*p*-Value
Stage	I	50.0	0.0	50.0	0.040
	II	31.3	31.3	37.5	
	III	33.3	33.3	33.3	
	IV	0.0	50.0	50.0	
Tumor size (mean, cm)		1.83 ± 1.04	3.04 ± 0.94	1.74 ± 1.10	0.029
Lymphovascular invasion	Negative	45.0	5.0	50.0	0.003
	Positive	23.1	46.2	30.8	
Lymph node metastasis	Negative	42.9	4.8	52.4	0.002
	Positive	25.0	50.0	25.0	
